# RNA G-quadruplexes and calcium ions synergistically induce Tau phase transition *in vitro*

**DOI:** 10.1016/j.jbc.2024.107971

**Published:** 2024-11-05

**Authors:** Yasushi Yabuki, Kazuya Matsuo, Ginji Komiya, Kenta Kudo, Karin Hori, Susumu Ikenoshita, Yasushi Kawata, Tomohiro Mizobata, Norifumi Shioda

**Affiliations:** 1 Institute of Molecular Embryology and Genetics (IMEG), Department of Genomic Neurology, Kumamoto University, Kumamoto, Japan; 2 Graduate School of Pharmaceutical Sciences, Kumamoto University, Kumamoto, Japan; 3 Department of Neurology, Graduate School of Medical Sciences, Kumamoto University, Kumamoto, Japan; 4 Department of Chemistry and Biotechnology, Graduate School of Engineering, Tottori University, Tottori, Japan

**Keywords:** RNA G-quadruplex, calcium ions, Tau, liquid–liquid phase separation, liquid–solid phase transition

## Abstract

Tau aggregation is a defining feature of neurodegenerative tauopathies, including Alzheimer’s disease, corticobasal degeneration, and frontotemporal dementia. This aggregation involves the liquid–liquid phase separation (LLPS) of Tau, followed by its sol–gel phase transition, representing a crucial step in aggregate formation both *in vitro* and *in vivo*. However, the precise cofactors influencing Tau phase transition and aggregation under physiological conditions (*e.g.,* ion concentration and temperature) remain unclear. In this study, we unveil that nucleic acid secondary structures, specifically RNA G-quadruplexes (rG4s), and calcium ions (Ca^2+^) synergistically facilitated the sol–gel phase transition of human Tau under mimic intracellular ion conditions (140 mM KCl, 15 mM NaCl, and 10 mM MgCl_2_) at 37 °C *in vitro*. In the presence of molecular crowding reagents, Tau formed stable liquid droplets through LLPS, maintaining fluidity for 24 h under physiological conditions. Notably, cell-derived RNA promoted Tau sol–gel phase transition, with rG4s emerging as a crucial factor. Surprisingly, polyanion heparin did not elicit a similar response, indicating a distinct mechanism not rooted in electrostatic interactions. Further exploration underscored the significance of Ca^2+^, which accumulate intracellularly during neurodegeneration, as additional cofactors in promoting Tau phase transition after 24 h. Importantly, our findings demonstrate that rG4s and Ca^2+^ synergistically enhance Tau phase transition within 1 h when introduced to Tau droplets. Moreover, rG4-Tau aggregates showed seeding ability in cells. In conclusion, our study illuminates the pivotal roles of rG4s and Ca^2+^ in promoting Tau aggregation under physiological conditions *in vitro*, offering insights into potential triggers for tauopathy.

The intrinsically disordered protein Tau is a neuronal microtubule-binding protein encoded by the *MAPT* gene (1). Tau mediates tubulin polymerization and microtubule stability, helping stabilize axons in the central nervous system (2, 3). In pathological conditions, Tau dissociates from microtubules and forms aggregates as neurofibrillary tangles, which may cause tauopathies such as Alzheimer’s disease (AD), corticobasal degeneration, and frontotemporal dementia (4, 5). Posttranslational modifications of Tau, such as phosphorylation and acetylation, bring about its detachment from microtubules *via* conformational changes, inducing its aggregation (3, 6). However, the mechanisms underlying the aggregation of dissociated Tau proteins remain elusive.

Liquid–liquid phase separation (LLPS) is a common phenomenon resulting from weak multivalent interactions, leading to the formation of droplets, hydrogels, and aggregates (7, 8, 9). Tau has a strong ability to undergo LLPS, which enhances physiological polymerization (10, 11). Tau protein LLPS can also initiate its liquid–solid phase transition, resulting in its aggregation (11, 12, 13, 14, 15). Polyanionic conditions, such as the presence of heparin or RNAs and hyperphosphorylation, promote Tau liquid–solid phase transition by enhancing LLPS (12, 14, 16, 17), suggesting that electrostatic interactions are critical for Tau condensates. However, *in vitro* experiments on Tau LLPS have been designed under nonphysiological conditions, such as low salt concentrations, room temperature, and high-frequency agitation, which do not accurately represent the intracellular environment; therefore, the triggering factors contributing to the Tau phase transition remain unclear.

RNA G-quadruplexes (rG4s) are quadruple-stranded nucleic acid secondary structures formed by contiguous guanines (18, 19). rG4s are involved in coordinating numerous stages of RNA metabolism, including RNA splicing, RNA processing and transport, and mRNA translation (20). rG4 itself has the potential to induce RNA LLPS, which may be associated with neurodegenerative diseases (8, 21, 22, 23). Importantly, we have shown that rG4s initiate the liquid–solid phase transition of prion-like proteins including α-synuclein (αSyn) and FMRpolyG, contributing to progressive neurodegeneration (24, 25).

Here, we show that rG4s and calcium ions (Ca^2+^) synergistically promote Tau phase transition under physiological conditions *in vitro*. Purified human Tau proteins formed liquid droplets *via* LLPS and remained in the fluid for 24 h. We found that rG4s and Ca^2+^ promoted Tau phase transition within 24 h. Furthermore, when both rG4s and Ca^2+^ were added to the Tau liquid droplets, a synergistic effect caused earlier phase transition and aggregation. These findings suggest that both rG4s and Ca^2+^ are important cofactors that promote the Tau phase transition.

## Results

### Cell-derived RNAs undergo Tau liquid–solid phase transition

**Figure 1:**
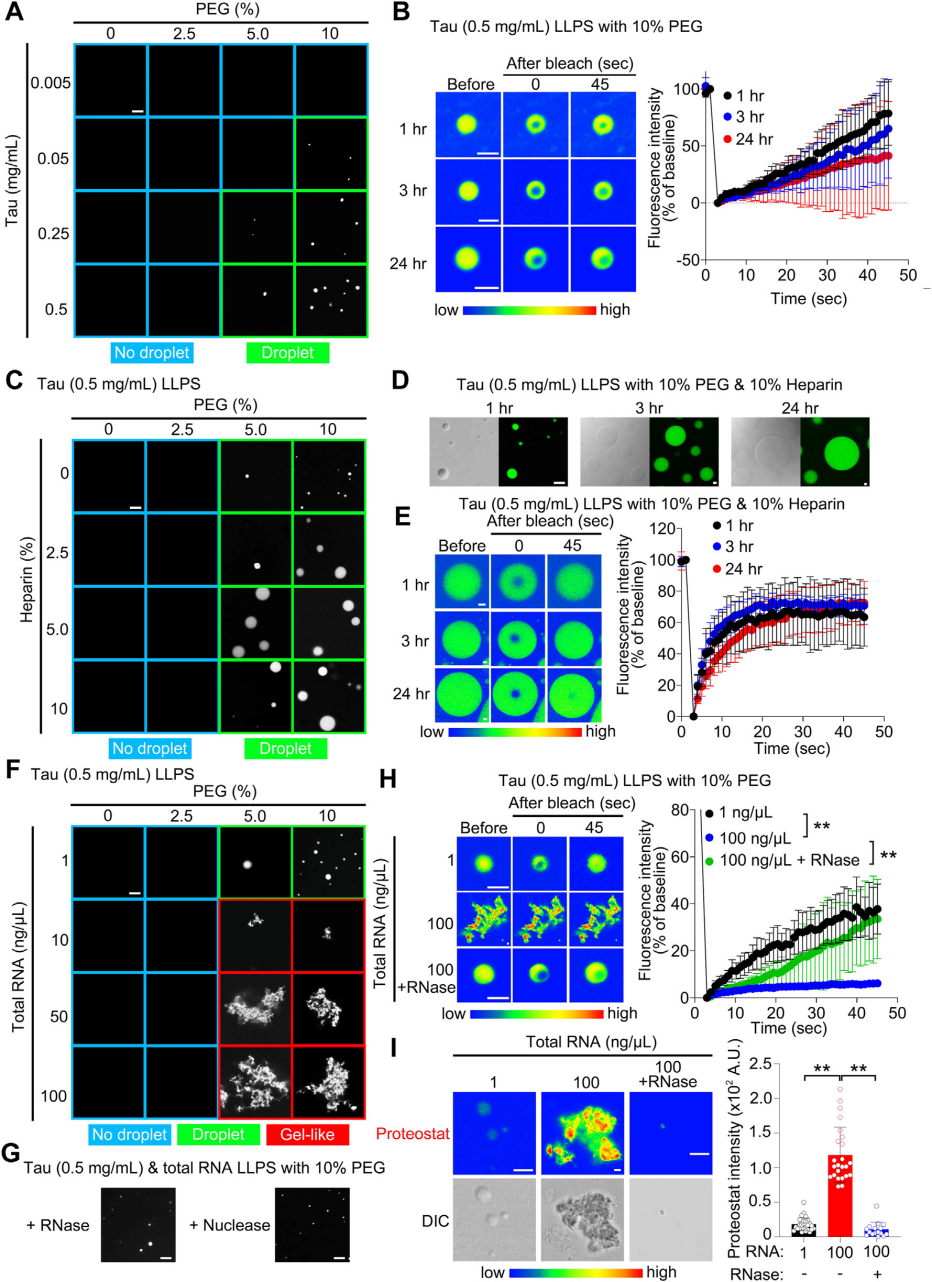
**RNA initiates Tau liquid–solid phase transition.***A,* representative images of *in vitro* Tau phase separation dependent on PEG and Tau protein concentration, when incubated at 37 °C for 1 h. The scale bar represents 5 μm. *B,* FRAP assays of Tau LLPS with 10% PEG at 1, 3, and 24 h after incubation. The scale bar represents 2 μm. n = 6 (1 h), n = 9 (3 h), and n = 9 (24 h). *C,* representative images of *in vitro* Tau (0.5 mg/ml) phase separation dependent on PEG and heparin concentration when incubated at 37 °C for 1 h. The scale bar represents 5 μm. *D,* representative images of *in vitro* Tau (0.5 mg/ml) phase separation with 10% PEG and 10% heparin at 1, 3, and 24 h after incubation. The scale bar represents 5 μm. *E,* FRAP assays of Tau LLPS with 10% PEG and 10% heparin at 1, 3, and 24 h after incubation. The scale bar represents 5 μm. n = 6 (1 h), n = 7 (3 h), and n = 6 (24 h). *F,* representative images of *in vitro* Tau (0.5 mg/ml) phase separation dependent on PEG and total RNA concentration at 1 h after incubation. The scale bar represents 5 μm. *G,* representative images of *in vitro* Tau (0.5 mg/ml) phase separation with 10% PEG and 100 ng/μl total RNA in the presence of RNase A (*right*) and nuclease (*left*) incubated at 37 °C for 1 h. The scale bar represents 5 μm. *H,* FRAP assays of Tau LLPS with 10% PEG and total RNA (1 or 100 ng/μl) with or without RNase A at 1 h after incubation. The scale bar represents 2 μm. n = 5 (1 ng/μl), n = 7 (100 ng/μl), and n = 6 (100 ng/μl + RNase A). *I,* analysis of Proteostat intensity within Tau LLPS with 10% PEG and total RNA (1 or 100 ng/μl) with or without RNase A at 1 h after incubation. The scale bar represents 2 μm. n = 30 (1 ng/μl), n = 24 (100 ng/μl), and n = 16 (100 ng/μl + RNase A). Data are presented as the mean ± SD. ∗∗*p* < 0.01 by two-way (*B*, *E*, and *H*) and one-way (*I*) analysis of variance with Bonferroni’s multiple comparisons test. LLPS, liquid–liquid phase separation.

First, we investigated whether purified human Tau undergoes LLPS *in vitro* using buffer conditions with physiologically relevant salt concentrations (140 mM KCl, 15 mM NaCl, and 10 mM MgCl_2_) at 37 °C, with the molecular crowding agent PEG. We confirmed that the purified human Tau was a monomer using western blotting (Fig. S1). To visualize Tau condensates using fluorescence, nonlabeled and fluorescein-labeled human Tau were mixed at a ratio of 1:1. Using buffer conditions with physiologically relevant salt concentrations, purified human Tau (0.5 mg/ml; 10.9 μM) underwent LLPS in the presence of > 5% PEG (w/v) (Fig. 1*A*). In the presence of 10% PEG (w/v), the threshold Tau concentration for LLPS (Fig. 1*A*) was lowered, indicating that its LLPS is dependent on molecular crowding conditions. To characterize the dynamic nature of the droplets, we performed a FRAP assay. No significant differences were observed in Tau droplets with 10% PEG (w/v) incubated at 37 °C from 1 to 24 h (Fig. 1*B*). We confirmed that Tau droplets induced sol-gel phase transition within 24 h under nonphysiological conditions (*e.g.,* low salt concentrations, room temperature [25 ± 2 °C]), and agitation (Fig. S2) (11, 14, 26). These results suggest that molecular crowding alone is not sufficient to induce Tau liquid–solid phase transition under physiological conditions (Fig. 1*B*).

Under the same physiological conditions, we investigated the effect of polyanionic reagents (*i.e.,* heparin and RNAs) on Tau LLPS. Tau droplets appeared to increase in size with increasing heparin concentration (Fig. 1*C*). Heparin (10%, w/v) seemed to result in the time-dependent enlargement of Tau droplets without affecting fluorescence recovery (Fig. 1, *D* and *E*). Tau droplets in 10% heparin showed rapid fluorescence recovery and maintained molecular diffusion inside condensates within 24 h, suggesting that polyanionic heparin may help buffer Tau inside droplets under physiological conditions. Conversely, total RNAs derived from neuro2a cells elicited Tau phase transition from a droplet to an aggregate state when incubated at 37 °C for 1 h (Fig. 1*F*). We confirmed that pretreatment with RNase A, or Cryonase Cold-active Nuclease, inhibited Tau liquid–solid phase transition (Fig. 1*G*). In addition, the application of RNA to Tau significantly reduced the fluorescence recovery of Tau condensates in FRAP and increased Proteostat intensity, which was prevented by pretreatment with RNase A (Fig. 1, *H* and *I*). We also confirmed the amorphous aggregates of Tau with total RNA by transmission electron microscopy (TEM), which did not form typical amyloid or fibers (Fig. S3). These results suggest that the properties of RNA other than anionic charge are involved in the process of Tau aggregation.

### rG4 promotes Tau liquid–solid phase transition

**Figure 2:**
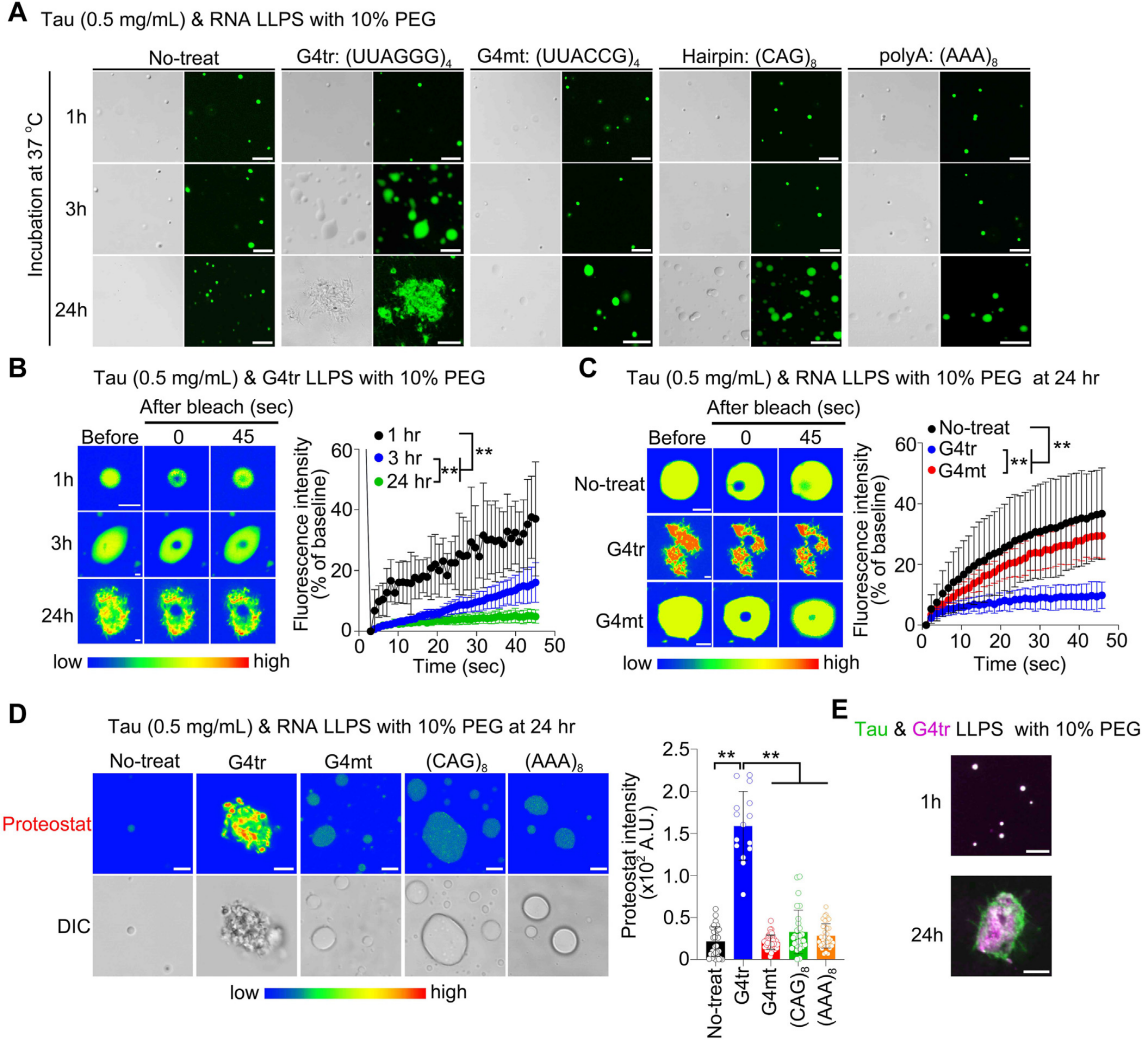
**rG4 accelerates Tau liquid–solid phase transition.***A,* representative images of *in vitro* Tau (0.5 mg/ml) phase separation with 10% PEG and 1 μM RNA nucleotides at 1, 3, and 24 h after incubation. The scale bar represents 5 μm. *B,* FRAP assays of Tau LLPS with 10% PEG and 1 μM G4tr at 1, 3, and 24 h after incubation. The scale bar represents 5 μm. n = 7 (1 h), n = 7 (3 h), and n = 6 (24 h). *C,* FRAP assays of Tau LLPS with 10% PEG in the presence of 1 μM G4tr or G4mt at 24 h after incubation. The scale bar represents 2 μm. n = 7 (1 h), n = 7 (3 h), and n = 6 (24 h). *D,* analysis of Proteostat intensity within Tau LLPS with 10% PEG and 1 μM RNA at 24 h after incubation. The scale bar represents 5 μm. n = 31 (untreated), n = 15 (G4tr), n = 40 (G4mt), n = 28 ((CAG)_8_), and n = 40 ((AAA)_8_). *E,* representative images of Tau (*green*) and G4tr (*magenta*) with 10% PEG incubated at 37 °C for 1 and 24 h. Data are presented as the mean ± SD. ∗∗*p* < 0.01 by two-way (*B* and *C*) and one-way (*D*) ANOVA with Bonferroni’s multiple comparisons test. rG4, RNA G-quadruplex.

Since rG4s have an important role in the liquid–solid phase transition of prion-like proteins *in vitro* and *in vivo*, including αSyn (24, 25), we investigated whether RNA structures are involved in Tau phase transition using various RNA oligonucleotides: G4tr, a typical rG4, telomeric repeat-containing RNA (TERRA) (UUAGGG)_4_ repeats; G4mt, a TERRA mutant that is unable to form rG4 (UUACCG)_4_ repeats, a mismatch-hairpin formed (CAG)_8_ repeats, and polyA; single-stranded (AAA)_8_ repeats (25). Similar to other prion-like proteins, G4tr triggered a Tau phase transition, leading to an aggregate-like state over time, whereas the other RNA structures showed an increase in liquid droplet size and remained in a liquid state similar to heparin (Fig. 2*A*). In the FRAP analysis, the fluorescence recovery of G4tr-treated Tau was markedly reduced in a time-dependent manner (Fig. 2*B*). After 24 h, the fluorescence recovery rate of Tau treated with G4tr was significantly lower than that of the untreated control and G4mt-treated Tau (Fig. 2*C*). Moreover, the intensity of Proteostat signals was significantly higher in G4tr-treated Tau than in Tau alone and non-G4 forming RNAs-treated Tau (Fig. 2*D*). We also confirmed the colocalization of cyanine5-labeled G4tr with Tau droplets after 1 h and Tau aggregates after 24 h (Fig. 2*E*).

To investigate whether Tau preferentially interacts with G4tr compared to other RNA structures, we performed surface plasmon resonance (SPR) binding analysis. The SPR analysis showed no significant differences in the affinity for any RNA structure, although the affinity of Tau for G4tr appeared to be slightly higher than that for other RNA structures (Table. 1; Fig. S4).

**Table 1:** Affinity of Tau for 24-mer RNA oligonucleotides as revealed by SPR

RNA	Ka (×10^2^ 1/M∗s)	Kd (×10^−4^ 1/s)	*K*_*D*_ (nM)
G4tr	293.5 ± 17.6	9.53 ± 2.84	32.1 ± 8.38
G4mt	264.5 ± 60.3	14.5 ± 5.78	52.5 ± 14.3
(CAG)_8_	319.3 ± 94.7	19.0 ± 7.28	57.3 ± 13.9
(AAA)_8_	256.8 ± 61.7	18.5 ± 6.93	71.7 ± 27.0

Tau concentration; 100, 200, 400, and 800 nM, Experimental buffer condition; 10 mM KCl in 50 mM Tris–HCl (pH = 7.4), Experimental temperature; room temperature (25 ± 2 °C). n = 4 per group. Data are presented as the mean ± SD.

Ka, association rate constant expressed in M^−1^s^−1^; Kd, dissociation rate constant expressed in s^−1^; *K*_*D*_, dissociation equilibrium (affinity) constant resulting from the ratio of Ka and Kd expressed in nM.

### Ca^2+^ facilitates Tau LLPS and rG4-induced Tau phase transition

**Figure 3:**
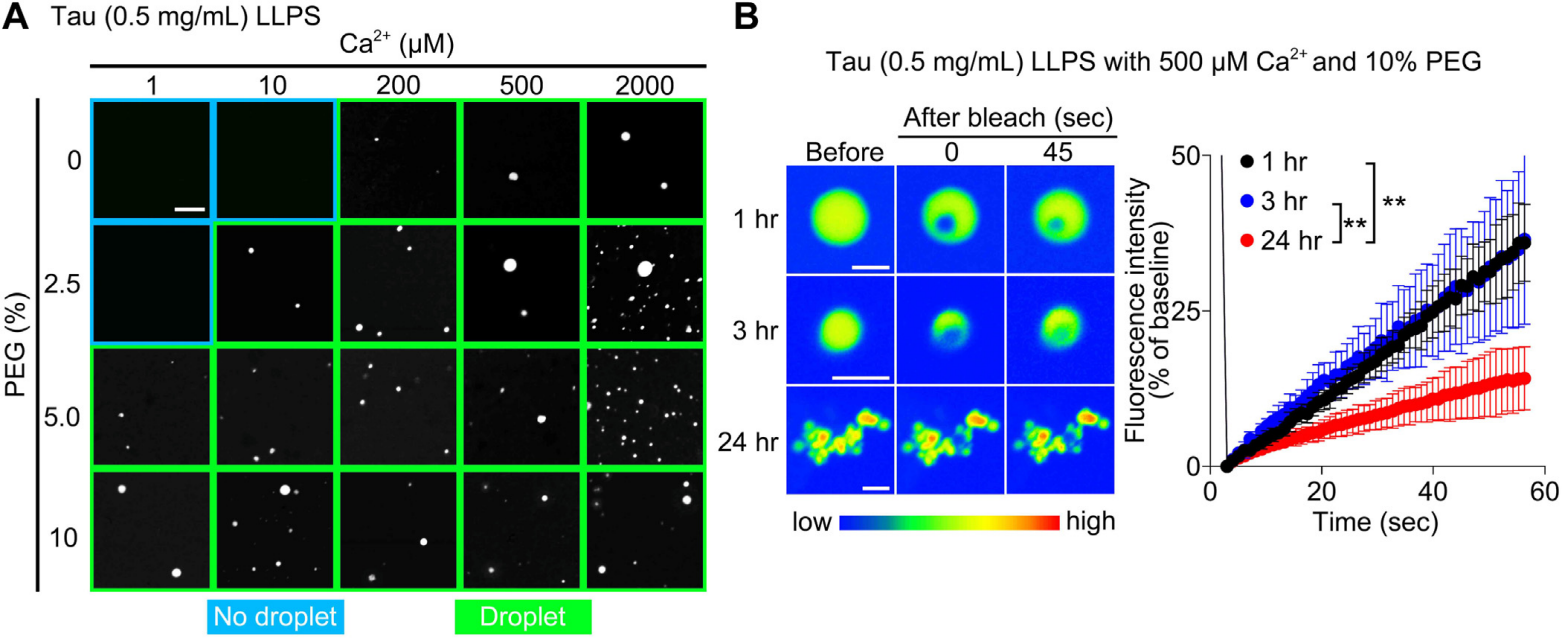
**Ca**^**2+**^**promotes Tau LLPS and liquid–solid phase transition.***A,* representative images of *in vitro* Tau (0.5 mg/ml) phase separation dependent on PEG and Ca^2+^ concentrations when incubated at 37 °C for 1 h. The scale bar represents 5 μm. *B,* FRAP assays of Tau LLPS with 10% PEG at 1, 3, and 24 h after incubation. The scale bar represents 2 μm. n = 9 per time point. Data are presented as the mean ± SD. ∗∗*p* < 0.01 by two-way (*B*) ANOVA with Bonferroni’s multiple comparisons test. Ca^2+^, calcium ions.

Persistent and excessive Ca^2+^ entry in neurons could be a major factor in the development of neurodegeneration, including tauopathies (27, 28). In addition, Tau aggregation is facilitated by divalent cations, including Ca^2+^ *in vitro* (29, 30). Thus, we investigated whether Ca^2+^ affects Tau LLPS- and rG4-induced Tau phase transitions *in vitro*. We found that Ca^2+^ significantly increased Tau LLPS within 1 h of incubation at 37 °C (Fig. 3*A*). Moreover, fluorescence recovery of fluorescein-labeled Tau in FRAP was markedly reduced in the presence of Ca^2+^ (500 μM) after 24 h (Fig. 3*B*), suggesting Ca^2+^ may be a key cofactor of Tau liquid–solid phase transition. SPR analysis showed no significant difference between the affinity of Tau for G4tr and G4mt in the presence of Ca^2+^ (500 μM) (Table. 2; Fig. S4).

**Table 2:** Affinity of Tau for G4tr and G4mt in the presence of 500 μM Ca^2+^ as revealed by SPR

RNA	Ka (×10^2^ 1/M∗s)	Kd (×10^−4^ 1/s)	*K*_*D*_ (nM)
G4tr	259.5 ± 25.4	18.5 ± 6.37	70.4 ± 22.4
G4mt	283.3 ± 71.7	22.3 ± 7.16	77.4 ± 6.73

Tau concentration; 100, 200, 400, and 800 nM, Experimental buffer condition; 10 mM KCl and 500 μM Ca^2+^ in 50 mM Tris–HCl (pH = 7.4), Experimental temperature; room temperature (25 ± 2 °C). n = 4 per group. Data are presented as the mean ± SD.

Ka, association rate constant expressed in M^−1^s^−1^; Kd, dissociation rate constant expressed in s^−1^; *K*_*D*_, dissociation equilibrium (affinity) constant resulting from the ratio of Ka and Kd expressed in nM; SPR, surface plasmon resonance.

**Figure 4:**
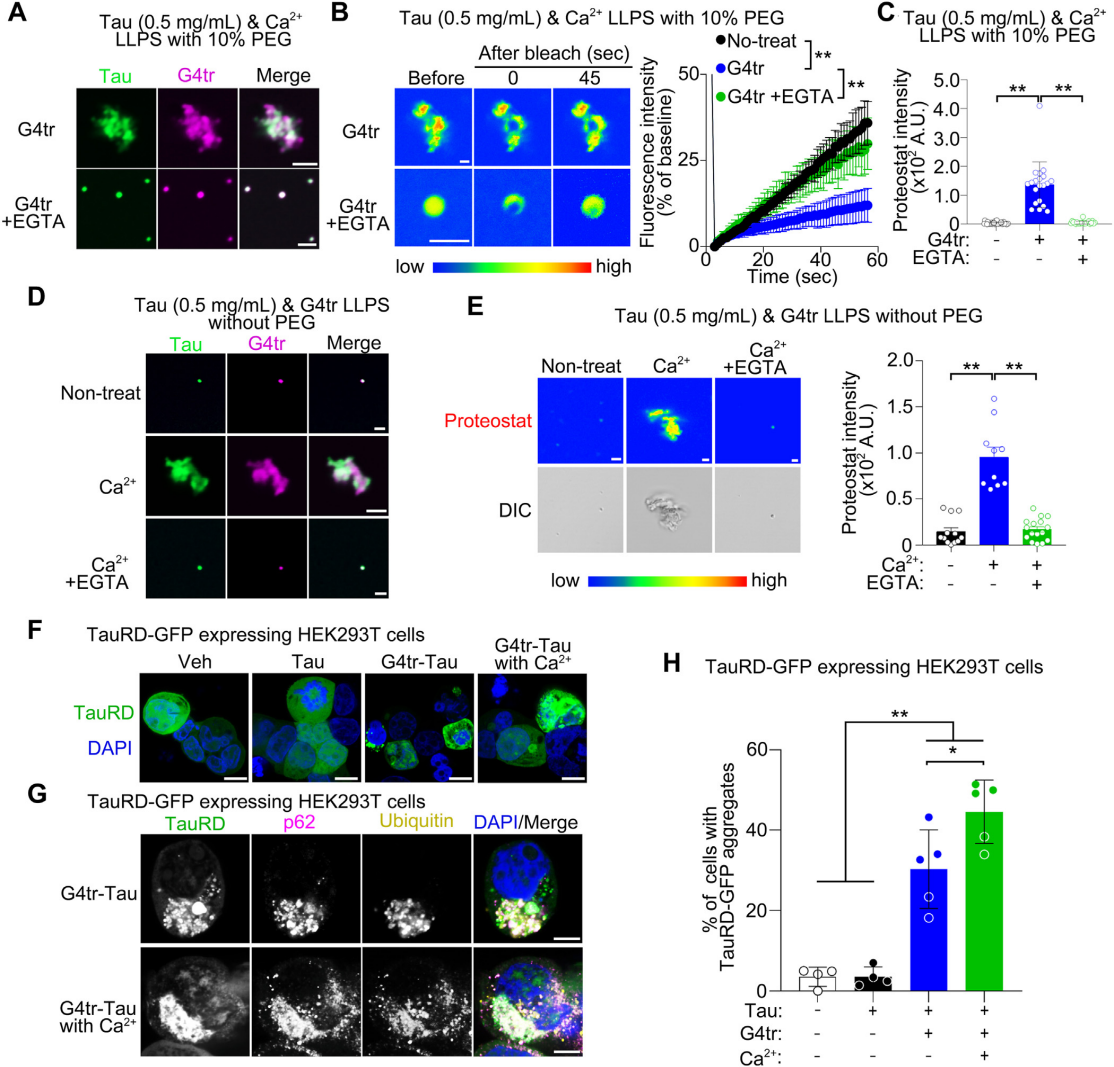
**Synergistic effect of Ca**^**2+**^**and G4tr on Tau liquid–solid phase transition.***A,* representative images of *in vitro* Tau (0.5 mg/ml; *green*) and G4tr (1 μM; *magenta*) phase separation in the presence of 10% PEG and 500 μM Ca^2+^ with or without 2.5 mM EGTA when incubated at 37 °C for 1 h. The scale bar represents 2 μm. *B,* FRAP assays of Tau LLPS in the presence of 10% PEG, 1 μM G4tr, and 500 μM Ca^2+^ with or without 2.5 mM EGTA when incubated at 37 °C for 1 h. The scale bar represents 2 μm. n = 9 (untreated), n = 8 (G4tr), and n = 6 (G4tr + EGTA). *C,* analysis of Proteostat intensity within Tau LLPS with 10% PEG, 1 μM G4tr, and 500 μM Ca^2+^ with or without 2.5 mM EGTA when incubated at 37 °C for 1 h. The scale bar represents 5 μm. n = 35 (untreated), n = 20 (G4tr), and n = 22 (G4tr + EGTA). *D,* representative images of *in vitro* Tau (0.5 mg/ml; *green*) and G4tr (1 μM; *magenta*) phase separation with or without 500 μM Ca^2+^ and 2.5 mM EGTA when incubated at 37 °C for 1 h. The scale bar represents 2 μm. *E,* analysis of Proteostat intensity within Tau LLPS with 1 μM G4tr in the presence or absence of 500 μM Ca^2+^ and 2.5 mM EGTA when incubated at 37 °C for 1 h. The scale bar represents 2 μm. n = 12 (G4tr), n = 10 (G4tr + Ca^2+^), and n = 16 (G4tr + Ca^2+^ + EGTA). *F,* representative images of TauRD-GFP (*green*) and DAPI (*blue*) in TauRD-GFP transfected HEK293T cells following treatment with *in vitro* Tau aggregates induced by G4tr in the presence or absence of Ca^2+^. The scale bars represents 10 μm. *G,* representative images of TauRD-GFP (*green*), p62 (*magenta*), ubiquitin (*yellow*), and DAPI (*blue*) in TauRD-GFP expressing HEK293T cells following G4tr-Tau with or without Ca^2+^. The scale bars represents 5 μm. *H,* the ratio of cells with p62- and ubiquitin-positive TauRD aggregates. n = 4 (vehicle), n = 4 (Tau), n = 5 (G4tr-Tau), and n = 5 (G4tr-Tau with Ca^2+^). Data are presented as the mean ± SD. ∗*p* < 0.05, ∗∗*p* < 0.01 by two-way (*B*) and one-way (*C*, *E*, and *H*) ANOVA with Bonferroni’s multiple comparisons test. DAPI, 4′,6-diamidino-2-phenylindole; Ca^2+^, calcium ions.

Notably, cyanine5-labeled G4tr coaggregated with fluorescein-labeled Tau in the presence of Ca^2+^ and 10% PEG (w/v) when incubated at 37 °C for 1 h (Fig. 4*A*). Ca^2+^ accelerated Tau liquid–solid phase transition by G4tr, resulting in a significant increase in Proteostat intensity and a decrease in Tau fluorescence recovery in FRAP, which was prevented by pretreatment with EGTA (Fig. 4, *B* and *C*). Consistent with that G4tr did not induce the liquid–solid phase transition of Tau within 1 h (Fig. 2, *A* and *B*), G4tr did not cause Tau aggregation at 1 h after chelating Ca^2+^ with EGTA. On the other hand, we confirmed that Ca^2+^ did not affect the G4mt-Tau LLPS with 10% PEG (w/v) (Fig. S5). Moreover, even under PEG-free conditions, Ca^2+^ induced the G4tr-Tau LLPS to shift to an aggregate-like state after 1 h, thereby significantly increasing the Proteostat intensity, whereas this effect was attenuated by pretreatment with EGTA (Fig. 4, *D* and *E*).

Finally, we investigated whether Tau aggregates induced by G4tr *in vitro* elicit aggregation of intracellularly expressing Tau in HEK293T cells, which are an epithelial-like cells isolated from the kidney of a patient. The ultrasonicated G4tr-Tau aggregates with or without Ca^2^ *in vitro* induced the aggregation of Tau repeat domain (TauRD)-GFP (31) formed in cells, which is positive for the aggregation markers p62 and ubiquitin (Fig. 4, *F* and *G*). In TauRD-GFP–expressing HEK293T cells, the ratio of cells with p62- and ubiquitin-positive GFP aggregates after treatment with G4tr-Tau with or without Ca^2+^ was significantly higher than that by vehicle or Tau alone. Moreover, the ratio in G4tr-Tau with Ca^2+^ was significantly higher than that in G4tr-Tau without Ca^2+^ (Fig. 4*H*). Thus, Tau aggregates induced by G4tr *in vitro* have intracellular seeding ability.

## Discussion

In the present study, we demonstrated that rG4 promoted the liquid-to-solid phase transition of Tau *in vitro*. Cell-derived total RNAs transform Tau droplets from a liquid to a gel-like state. We found that rG4 in RNAs enables the phase transition of Tau. Moreover, Ca^2+^ can facilitate Tau phase separation and aggregation, thereby accelerating rG4-induced Tau phase transition. Taken together, these results suggest that rG4 and Ca^2+^ may be key cofactors in Tau aggregation.

Although our study showed heparin-enlarged Tau droplets without phase transition under physiological conditions (Fig. 1), previous reports have demonstrated that heparin induces Tau aggregation *in vitro* under nonphysiological conditions (14, 17, 26, 32). Elevated cationic strength (>50 mM NaCl) significantly attenuates heparin-induced Tau aggregation (33, 34, 35), suggesting that decreased electrostatic interactions by mimicking intracellular ion conditions (140 mM KCl, 15 mM NaCl, and 10 mM MgCl_2_) may prevent the effect of heparin on Tau aggregation (33). In addition, the aggregated form of Tau induced by heparin differs from that in tauopathies, including AD and frontotemporal dementia (36, 37). Moreover, heparin-induced Tau aggregates fail to initiate Tau aggregation in primary neurons and mouse brains (38). These data suggest that the aggregation of Tau by polyanions may be artificial and not occur in cells.

Although rG4 facilitated the Tau liquid-to-solid phase transition (Fig. 2), there were no dramatic changes in its affinity for Tau compared to that of other structural RNAs (Table 1; Fig. S4). However, the fit was not of particularly good quality and the SPR data in the present study should be considered qualitatively (Fig. S4*C*). The process of LLPS in prion-like RNA-binding proteins (RBPs), including Tau, is differentially influenced by the type of RNA (39, 40, 41, 42), suggesting that the specific domain of RBPs to which the RNA binds and the characteristics of the RNA, such as length and structure, rather than the binding affinity of the RNA, intricately affect Tau LLPS. For example, fused in sarcoma, a representative amyotrophic lateral sclerosis–linked RBP, undergoes a liquid-to-solid phase transition by adding rG4, but not randomized RNAs (43). Other amyotrophic lateral sclerosis–linked RBP and TAR DNA-binding protein 43 condensates are buffered by the addition of tRNA, whereas nuclear-enriched abundant transcript 1 lncRNA, which can form rG4, facilitates TAR DNA-binding protein 43 LLPS (40, 44). Moreover, rG4 within prion mRNA may contribute to destabilizing the structure of prion proteins and trigger their conversion to an infectious form (45). We have also demonstrated that rG4 may change the structure of αSyn by binding to its N-terminus, leading to αSyn aggregation and in turn neurodegeneration in mouse neurons (25). The effect of rG4 on the structure of prion-like RBPs and the mechanism by which rG4 initiates aggregation are yet to be resolved, suggesting that rG4 can alter these properties.

In addition, we found that Ca^2+^ accelerated rG4-induced Tau phase transition (Figure 3, Figure 4). Consistent with our observations, previous reports have indicated that Ca^2+^ can accelerate the fibril formation of Tau *in vitro* (30, 46). Another bivalent cation, Zn^2+^, also facilitates Tau LLPS and fibrilization by binding to Cys residues present in the microtubule-binding repeat domain (Cys-291 and Cys-322) (30, 47), suggesting that Ca^2+^ binding to Cys in the microtubule-binding repeat domain may be associated with Tau aggregation (30). Furthermore, we here showed that Tau aggregates induced by G4tr *in vitro* showed seeding ability in TauRD-expressing cells, and the ratio of aggregates in the presence of Ca^2+^ was significantly higher than that in the absence of Ca^2+^ (Fig. 4, *F–H*). The difference in the seeding ability of G4tr-Tau aggregates with or without Ca^2+^ may be due to variances in their aggregate structure. Further studies are required to uncover the mechanism underlying rG4- and Ca^2+^-induced Tau aggregation, such as the identification of the binding sites of rG4 and Ca^2+^ to Tau using cryo-EM and nuclear magnetic resonance spectrometry.

Impaired RNA metabolism has been recognized as a common event in neurodegeneration, especially the formation of RNA granules, termed stress granules (SGs), which appear to be particularly relevant to neurodegenerative diseases, including tauopathy (48). Cellular stress, such as treatment with MG-132 or arsenite, triggers the formation of T-cell intracytoplasmic antigen 1-positive SGs; in turn, Tau is recruited to SGs in Tau-overexpressing mouse neuronal cell lines and primary neurons, resulting in an increase in misfolded and phosphorylated Tau (49, 50). T-cell intracytoplasmic antigen 1 knockdown attenuates Tau aggregation, neuronal death, and memory deficits in mouse models expressing human mutated Tau (P301L or P301S) (50, 51), suggesting that the recruitment of Tau into SGs may trigger aggregation. Kharel *et al.* reported that various cellular stressors markedly elevated rG4 levels in human cells (52). Moreover, we previously demonstrated that rG4s are highly enriched in SGs and organize T-cell intracytoplasmic antigen 1-positive SGs assemblies in primary mouse neurons (23). These observations suggest that coaggregation of rG4s and Tau in SGs under cellular stress conditions contributes to neurodegeneration. There have also been an increasing number of studies focusing on the role of rG4 in human aging and neurodegenerative diseases (53, 54). In AD neurons, the accumulation of rG4 structures is observed in lamina-associated domains (55). Importantly, Kallweit *et al.* demonstrated a significant positive correlation between rG4 accumulation and the Braak stage in the outer molecular layer region of the AD brain (56). They also indicated that neurofibrillary tangles contain rG4s in AD neurons (56), suggesting that rG4s may contribute to Tau aggregation in the human brain.

In conclusion, rG4 and Ca^2+^ synergistically induced Tau aggregation *in vitro*. The application of heparin and other structured RNAs failed to transition Tau coacervates to an aggregate-like state, indicating that the rG4-induced Tau phase transition does not occur through electrostatic interactions. Since rG4 can initiate the phase transition of prion-like proteins including αSyn and FMRpolyG (24, 25), rG4 may be a common key factor in neurodegeneration.

## Experimental procedures

### Purification and fluorescein-labeling of Tau proteins

The pET23a-human Tau441 plasmid was expressed in *Escherichia coli* BLR (DE3) cells harboring an overproducing plasmid and purified as previously described (32). Briefly, the bacterial lysate obtained by ultrasonication was incubated at 80 °C for 10 min, immediately cooled on ice, and centrifuged to remove insoluble matter. Streptomycin sulfate (final concentration, 2.5%, w/v) was added to the supernatant to precipitate the nucleic acids. After centrifugation, the supernatant was dialyzed overnight against a purification buffer (50 mM Tris–HCl buffer, 2 mM EDTA-Na, 2 mM DTT, pH 7.8). The dialysate was fractionated using cation-exchange chromatography (HiTrap SP HP; GE Healthcare). Bound samples were eluted by applying a linear gradient of 0 to 0.5 M NaCl performed on an AKTA-explorer system (GE Healthcare) at 4 °C with high salt purification buffer (50 mM Tris–HCl buffer, 2 mM EDTA-Na, 2 mM DTT, 4M NaCl, pH 7.8). Samples were desalted and stored in a lyophilized state at 4 °C until being assayed. For fluorescence detection, recombinant Tau proteins were labeled with fluorescein using the Fluorescein Labeling Kit-NH2, according to the manufacturer’s instructions (Dojindo Molecular Technologies Inc.).

### RNA oligonucleotides

The RNA oligonucleotides were synthesized by Hokkaido System Science: nonlabeled and biotin-labeled: r (UUAGGG)_4_, r (UUACCG)_4_, r (CAG)_8_, and r (AAA)_8_; cyanine5-labeled: r (UUAGGG)_4_ and r (UUACCG)_4_. Each RNA was resolved in RNase-free water at a concentration of 100 μM.

### *In vitro* LLPS, Proteostat, and FRAP assay

Nonlabeled and fluorescein-labeled human Tau (0.005–0.5 mg/ml; 0.109–10.9 μM) were prepared in the LLPS assay buffer: 50 mM Tris–HCl buffer (pH 7.4) containing 140 mM KCl, 15 mM NaCl, 10 mM MgCl_2_, 0 to 10% PEG8000 (w/v) (MP Biomedicals), and 1 unit/μl RNase inhibitor (TOYOBO), in the presence or absence of 0.001 to 2 mM CaCl_2_ and 2.5 mM EGTA. Heparin (0–10%, w/v) (NACALAI TESQUE, INC.), total RNA (1–100 ng/μl), or synthesized 24-mer RNA oligonucleotides (1 μM) were added and incubated at 37 °C for 1, 3, and 24 h. Total RNA was extracted using an RNeasy Mini Kit (Qiagen) from intact Neuro-2a cells. Some samples with total RNA (in the absence of an RNase inhibitor) were treated with RNase A (8 μg/μl; Qiagen) or Cryonase Cold-active Nuclease (1.6 U/μl; TAKARA) for 1 h before adding the Tau protein. Samples were incubated at 37 °C for 1, 3, or 24 h.

Tau LLPS was also investigated under nonphysiological conditions (25 mM Hepes buffer pH 7.4 in 10 mM NaCl and 10% PEG (w/v) at room temperature (25 ± 2 °C); 30 mM Tris–HCl buffer pH 7.5 in 10 mM NaCl, 15% PEG (w/v), and 3% heparin (w/v) with 200 rpm agitation at 37 °C; 20 mM Hepes buffer pH 7.0 in 17% heparin (w/v) at room temperature (25 ± 2 °C)) at 1 and 24 h after incubation according to previous reports (11, 14, 26). The reacted solutions were mounted on glass slides using a 0.12 nm spacer (Sigma-Aldrich) and a coverslip. For fluorescence observations, nonlabeled human Tau proteins were mixed with fluorescein-labeled proteins at a ratio of 1:1. To assess the aggregated form of Tau, 2% (v/v) Proteostat reagent (Enzo Life Sciences) was added to the LLPS assay buffer. Differential interference contrast images were obtained using a TCS SP8 confocal microscope (Leica Microsystems). Protein intensities were analyzed using the LAS X system (Leica Microsystems). For the FRAP assay, samples were photobleached with 50% laser power, and time-lapse images were recorded every 1 s using a Zeiss Objective Plan-Apochromat 63 × /1.4 oil DIC M27 to track photorecovery behavior using an LSM900 microscopy system (Carl Zeiss).

### SPR-binding assays

SPR experiments were performed using OpenSPR (Nicoya) as previously reported (25). Briefly, biotin-labeled RNA oligonucleotides were fixed with a sensor chip using a biotin-streptavidin sensor kit (Nicoya), and Tau in running buffer (50 mM Tris–HCl pH 7.4 and 10 mM KCl) was streamed over the sensor chip to allow interaction. After ligand signal stabilization, the running buffer flowed at a rate of 20 μl/min for 5 min to collect dissociation data. Binding kinetic parameters were obtained by fitting the curve to a one-to-one binding model using TraceDrawer software (Ridgeview Instruments; https://tracedrawer.com/).

### TEM analysis

TEM image for total RNA-induced Tau aggregates was obtained using an HT7700 system (Hitachi High-Tech Corporation) operating at 80 kV. Samples of 10 μl were applied to a carbon-reinforced collodion film coated on copper mesh (400 mesh; Nisshin EM) and incubated for 5 min. The mesh was briefly washed with 5 μl of distilled water, followed by the application of 5 μl of 10% EM-stainer solution (v/v) (Nissin-EM) for 2 min. After a brief wash with 5 μl of distilled water, the sample was allowed to dry overnight before imaging.

### Western blotting

Western blot analyses were performed as previously described (24, 25). Briefly, the purification of Tau (0.25 and 0.50 mg/ml) were then boiled for 3 min in Laemmli's sample buffer. Samples were loaded onto SDS-polyacrylamide gels. For Coomassie brilliant blue staining, the above-loaded gel was incubated with EzStain AQua (ATTO) for 30 min at room temperature (25 ± 2 °C). After several washing with distilled water, the stained gel was scanned using a scanner (Canon). For immunoblotting, loaded proteins in gel were transferred to Immobilon polyvinylidene difluoride membranes, the membranes were blocked and incubated with anti-4R Tau (1:2000; 3E8-1A6; FUJIFILM Wako Pure Chemical Corporation), or anti-3R Tau (1:2000; 2A1-1F4; FUJIFILM Wako Pure Chemical Corporation) at 4 °C overnight. Blots were developed using an ECL immunoblotting detection system (Amersham Biosciences), and signals were detected using FUSION SOLO S (Viber-Lourmat).

### Cell culture, transfection, and Tau aggregates treatment

HEK293T cells (CRL-3216, American Type Culture Collection) were maintained at 37 °C and 5% CO_2_ in Dulbecco's modified Eagle's medium (Sigma-Aldrich), 10% heat-inactivated fetal bovine serum (v/v) (Sigma-Aldrich), and 1% penicillin/streptomycin (v/v) (Gibco). Transient transfection of pCAG-TauRD-GFP to HEK293T cells was performed using Lipofectamine 3000 according to the manufacturer’s instructions. After transfection, HEK293T cells were cultured in Dulbecco's modified Eagle's medium (Sigma-Aldrich) without fetal bovine serum. Transduction of ultrasonicated G4tr-Tau aggregates at 0.1 and 0.4 μM with HEK293T cells was performed using Lipofectamine 2000 as described previously (31). Nonlabeled human Tau (0.5 mg/ml; 10.9 μM) with or without G4tr (1 μM) were prepared in the filter-sterilized LLPS assay buffer (vehicle): 50 mM Tris–HCl buffer (pH 7.4) containing 140 mM KCl, 15 mM NaCl, 10 mM MgCl_2_, 10% PEG8000 (MP Biomedicals), and 1 unit/μl RNase inhibitor (TOYOBO), in the presence or absence of 500 μM CaCl_2_. Tau with or without G4tr was incubated at 37 °C for 24 h in the absence of 500 μM Ca^2+^. Under the condition in the presence of 500 μM Ca^2+^, Tau with G4tr was incubated at 37 °C for 1 h. After incubation, each Tau was sonicated using ultrasonic homogenizer sonifier SFX250 (Branson) for three times at 45% amplitude (on/off cycle = 10 s/10 s, 12 cycles; at 25 °C) and then applicated to TauRD-GFP expressing HEK293T cells. Forty-eight hours after incubation, cells were fixed with 4% paraformaldehyde for immunohistochemical analysis.

### Immunohistochemical analysis

Immunohistochemistry was performed as previously described (24, 25). Fixed cells with 4% paraformaldehyde (w/v) were washed with 1 × PBS, permeabilized with 0.1% Triton X-100 (v/v), and incubated with 1% bovine serum albumin and 0.1 Triton X-100 in 1 × PBS. One hour after blocking, samples were treated with primary antibodies as anti-p62 (1:1000; GP62-C, PROGEN Biotechnik) and antiubiquitin (1:1000; ZO458, Dako), which are a marker of aggregates, overnight at 4 °C. After several washing, cells were incubated overnight with the following secondary antibodies Alexa 594–conjugated goat anti-guinea pig (1:1000; A11076, Invitrogen) and Cy5-conjugated donkey anti-rabbit (1:1000; 711-175-152, Jackson ImmunoResearch). After several washes and 4′,6-diamidino-2-phenylindole staining, cells were mounted in Vectashield (Vector Laboratories, Inc.). Immunofluorescence images were obtained using TCS SP8 confocal microscopy system with a 63 × oil objective (NA 1.4) without z-stack (Leica Microsystems). For quantification, the number of GFP-positive cells from five randomly selected images was defined as one sample (comprising approximately 50 cells), and four to five samples were analyzed per group. Immunofluorescence images were taken from two independent experiments. The ratio of cells containing TauRD-GFP aggregates was determined by dividing the number of cells with p62- and ubiquitin-positive GFP aggregates by the total number of GFP-positive cells.

### Statistical analysis

Data are presented as the mean ± SD. The statistical significance of differences among groups was tested by one-way (Proteostat intensity and ratio of cells with TauRD-GFP aggregates) or two-way ANOVA (FRAP assay) with post hoc Bonferroni’s multiple comparison test. *p* < 0.05 represented a statistically significant difference. All statistical analyses were performed using the GraphPad Prism 7 software (GraphPad Software; https://www.graphpad.com/features).

## Data availability

All data needed to evaluate the conclusions in the paper are present in the article. Additional data related to this paper may be requested from the corresponding author upon reasonable request.

## Supporting information

This article contains supporting information.

## Conflict of interests

The authors declare that they have no conflicts of interest with the contents of this article.
