# Supplementary material for: RNA G-quadruplexes and calcium ions synergistically induce Tau phase transition in vitro
Source: J Biol Chem. 2024 Nov 5;300(12):107971. doi: 10.1016/j.jbc.2024.107971 (PMC11648224; doi:10.1016/j.jbc.2024.107971)
Supplement: Supplemental Figure S1–S5 [file mmc1.docx]

**
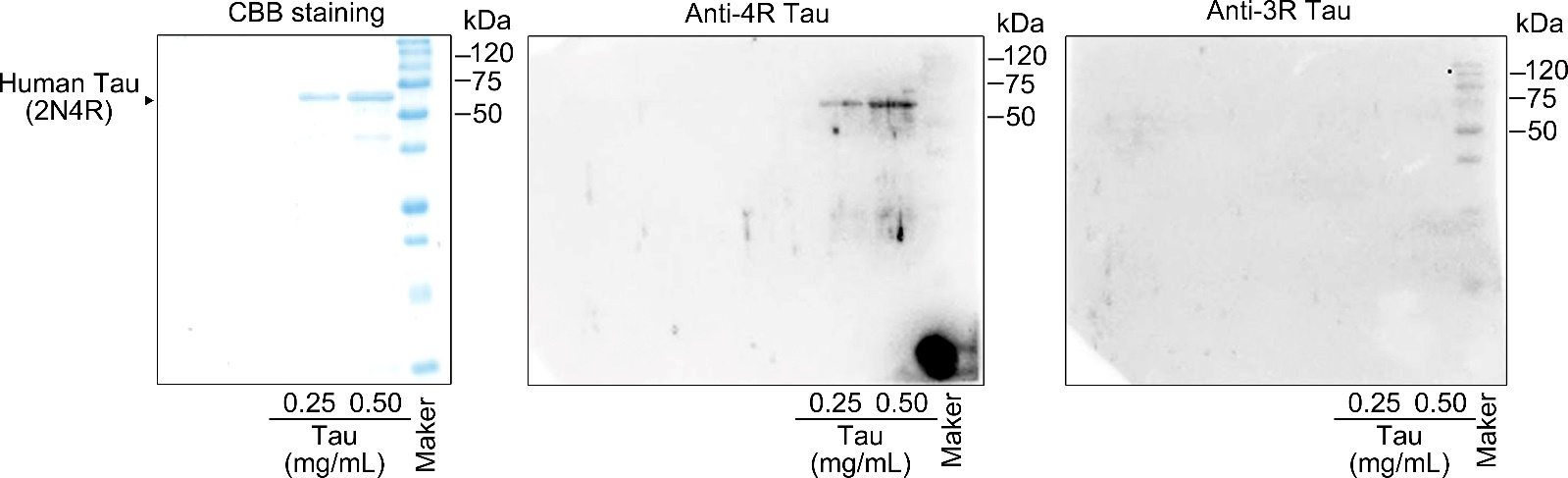
**

**Fig. S1 The verification of purified Tau.**

Representative images of the purified Tau detected by CBB staining (left), anti-4R Tau antibody (middle), and anti-3R Tau (right). Full length of Tau (2N4R) was purified as a monomer.

**
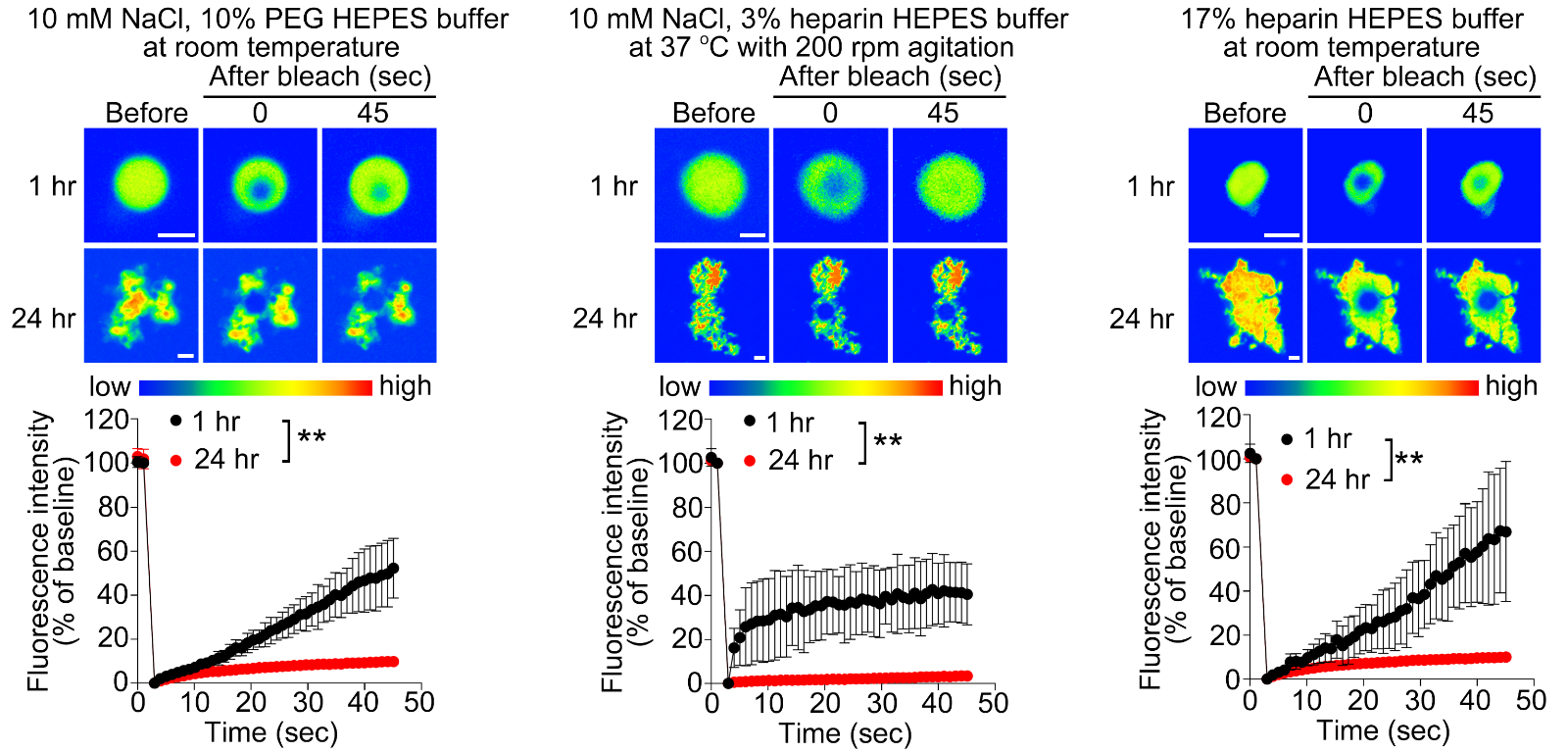
Fig. S2 Tau LLPS at non-physiological conditions.**

FRAP assays of Tau LLPS incubated for 1 or 24 h at non-physiological conditions: 25 mM HEPES buffer pH 7.4 with 10 mM NaCl and 10% PEG at room temperature (25±2℃) (right); 30 mM Tris-HCl buffer pH 7.5 with 10 mM NaCl, 15% PEG, and 3% heparin with 200 rpm agitation at 37℃ (center); 20 mM HEPES buffer pH 7.0 with 17% heparin at room temperature (25±2℃) (left). n = 5–7 per group per time point. Data are presented as the mean ± standard division. ***P* < 0.01 by two-way analysis of variance with Bonferroni’s multiple comparisons test.


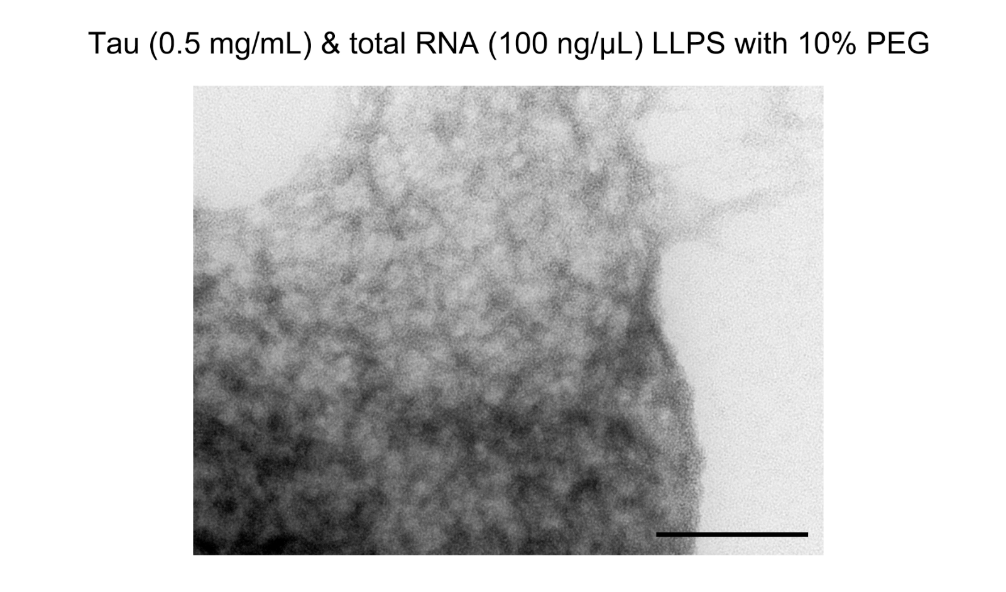


**Fig. S3 Transmission electron microscopy images for Tau with total RNA.**

Tau (0.5 mg/mL) was incubated with total RNA (100 ng/μL) under mimic intracellular ion conditions (140 mM KCl, 15 mM NaCl, and 10 mM MgCl_2_) at 37℃, with 10% PEG for one hour. Scale bars, 200 nm.


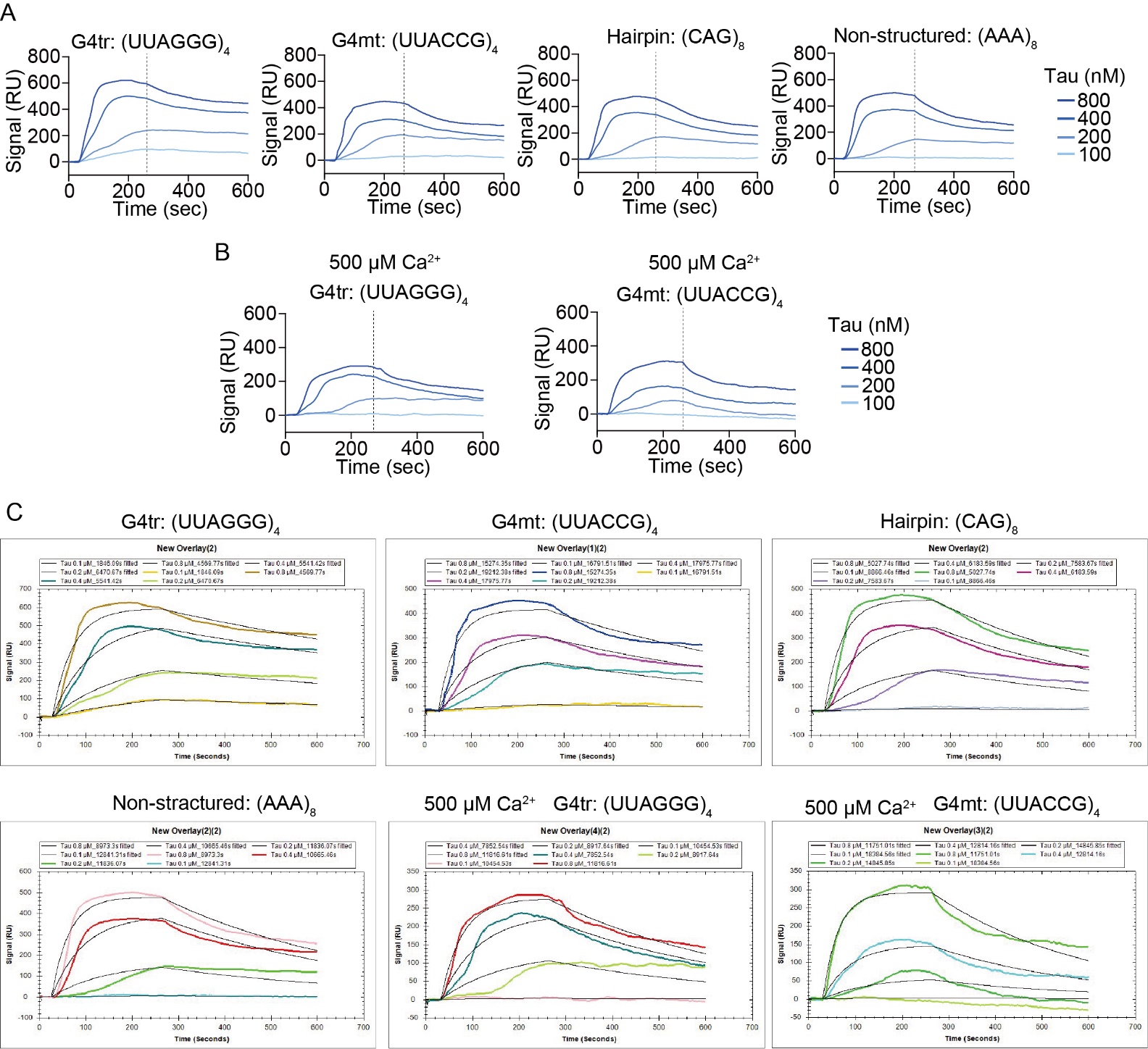
**Fig. S4 SPR sensorgrams for the interaction of Tau with 24-mer RNA oligonucleotides.**

The SPR sensorgrams used in the analysis for Table 1 (A) and Table 2 (B). A black dotted line showed the dissociation timing. (C) The SPR sensorgram displaying a fitting curve for the interaction of Tau with each RNA. The fitting curves are shown by the black line.


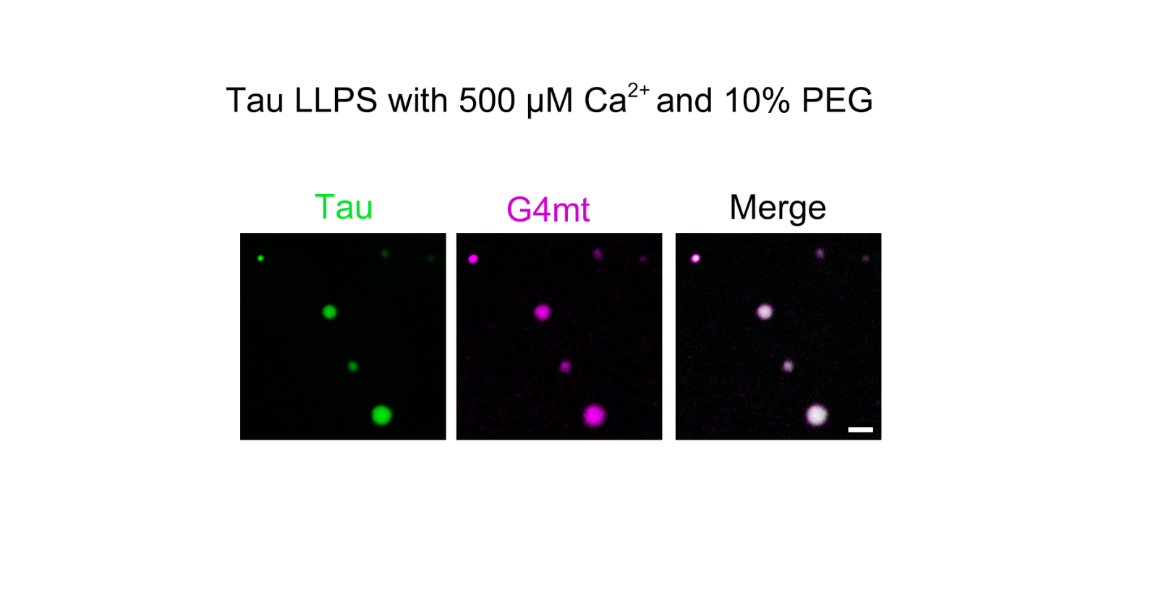


**Fig. S5 Ca^2+^ does not affect Tau and G4mt condensates.**

Representative images of *in vitro* Tau (0.5 mg/mL; green) and G4mt (1 μM; magenta) phase separation in the presence of 10% PEG and 500 μM Ca^2+^ when incubated at 37℃ for 1 h. Scale bar, 2 μm.
